# Information-incorporated sparse convex clustering for disease subtyping

**DOI:** 10.1093/bioinformatics/btad417

**Published:** 2023-06-29

**Authors:** Xiaoyu Zhang, Ching-Ti Liu

**Affiliations:** Department of Biostatistics, Boston University School of Public Health, Boston, MA 02118, United States; Department of Biostatistics, Boston University School of Public Health, Boston, MA 02118, United States

## Abstract

**Motivation:**

Heterogeneity in human diseases presents clinical challenges in accurate disease characterization and treatment. Recently available high throughput multi-omics data may offer a great opportunity to explore the underlying mechanisms of diseases and improve disease heterogeneity assessment throughout the treatment course. In addition, increasingly accumulated data from existing literature may be informative about disease subtyping. However, the existing clustering procedures, such as Sparse Convex Clustering (SCC), cannot directly utilize the prior information even though SCC produces stable clusters.

**Results:**

We develop a clustering procedure, information-incorporated Sparse Convex Clustering, to respond to the need for disease subtyping in precision medicine. Utilizing the text mining approach, the proposed method leverages the existing information from previously published studies through a group lasso penalty to improve disease subtyping and biomarker identification. The proposed method allows taking heterogeneous information, such as multi-omics data. We conduct simulation studies under several scenarios with various accuracy of the prior information to evaluate the performance of our method. The proposed method outperforms other clustering methods, such as SCC, K-means, Sparse K-means, iCluster+, and Bayesian Consensus Clustering. In addition, the proposed method generates more accurate disease subtypes and identifies important biomarkers for future studies in real data analysis of breast and lung cancer-related omics data. In conclusion, we present an information-incorporated clustering procedure that allows coherent pattern discovery and feature selection.

**Availability and implementation:**

The code is available upon request.

## 1 Introduction

Disease heterogeneity is common in many complex diseases, such as cancer. As a result, patients with a shared diagnosis may have different clinical responses to the same treatment. Such heterogeneity could be due to disease subtypes with distinct physiological processes. Therefore, accurate identification of these disease subtypes becomes critical for precision medicine. Breast cancer, e.g. four molecular subtypes (i.e. ER+/luminal-like, basal-like, HER2-enriched, and normal-like) with distinct clinical responses to treatments have been identified through gene expression data (Perou *et al.* 2000, van 't Veer *et al.* 2002). The Cancer Genome Atlas Networks (TCGA) group further demonstrated these four subtypes of breast cancer via multi-omics datasets, including genomic DNA copy number arrays, DNA methylation, exome sequencing, messenger RNA arrays, and microRNA sequencing (Cancer Genome Atlas Network 2012). Using omics datasets to identify disease subtypes has been extensively studied in many complex diseases beyond breast cancer, such as leukemia (Golub *et al.* 1999, Bullinger *et al.* 2004), lymphoma (Alizadeh *et al.* 2000, Rosenwald *et al.* 2002), colorectal cancer (Mo *et al.* 2013, Sadanandam *et al.* 2013), and Alzheimer’s disease (Bredesen 2015, Meng *et al.* 2022).

Clustering, an unsupervised machine learning method, has been widely applied to the disease subtyping problem (Zhang *et al.* 2022). However, most popular clustering algorithms, such as K-means, hierarchical clustering, and model-based clustering, suffer from instability (i.e. a different initialization or a slight change in data may yield different results) due to their nonconvex objective functions. Convex clustering thus has been proposed to address this issue (Pelckmans *et al.* 2005, Hocking *et al.* 2011, Lindsten *et al.* 2011, Chi and Lange 2015). In addition, convex clustering enjoys an appealing property obtaining a globally optimal solution. Recently, sparse convex clustering (SCC) was developed as an extension of convex clustering to efficiently deal with large datasets and select driving features simultaneously (Wang *et al.* 2018). This newly acquired property is essential and much needed when analyzing high-throughput omics datasets for disease subtyping.

Nevertheless, other noisy patterns within omics datasets may compromise the power to identify clinically meaningful subtypes. For example, in genetics studies, clusters could be driven by sex-related genes, age-related genes, or other unknown confounders-related genes instead of disease-related genes that interest us (Meng *et al.* 2022). Here, noisy clusters refer to those driven by nondisease-related biomarkers, as illustrated in Supplementary Fig. S1. Supplementary Figure S1 is a heatmap for a simulated data matrix with *n* = 60 samples by *p* = 100 features. We aim to find coherent sample clusters associated with the disease of interest. Supplementary Figure S1 has two types of clusters: one defined by features 1–30 and the other by features 31–50. The signal strength of the first type is slightly stronger than the second type. If the disease of interest is only associated with the first type of clusters, then identifying them would be challenging since most existing clustering methods would be compromised by the second type of clusters (i.e. noisy clusters) here. Similar scenarios have also been discussed by Gaynor and Bair (2017) and Nowak and Tibshirani (2008).

Incorporating clinical information can facilitate the identification of disease-related subtypes (Bair and Tibshirani 2004, Meng *et al.* 2022). Previous studies have shown that the incorporation of prior information can improve model performance in various areas, such as variable selection for generalized linear models (Jiang *et al.* 2016), identifying gene-environment interactions (Wang *et al.* 2019), and network analysis of gene expression data (Wu *et al.* 2023). However, direct clinical information for samples may not always be available, and we may alternatively leverage historical data, such as published studies, to help identify disease-related subtypes. One common approach to extracting information from published studies is to mine biomedical literature from publicly available databases, such as PubMed, Embase, and the Cochrane Library. There are several benefits of doing that. First, these databases comprise a growing number of mostly independent studies. The information is regularly updated and relatively reliable. Second, it is more cost-effective than collecting additional data by the researchers themselves. Therefore, we propose a novel clustering procedure incorporating prior information via text mining on PubMed.

In this article, we extend sparse convex clustering (Wang *et al.* 2018) and propose an information-incorporated sparse convex clustering (iSCC) procedure to identify disease-related subtypes and their driven features using multi-omics datasets. The rest of the article is structured as follows. We first present the proposed method and the implemented algorithm. We then conduct simulation studies and real data applications to compare the performance of our proposed method with other alternative methods. Finally, we conclude this article with a general discussion.

## 2 Materials and methods

In this section, we will present our proposed procedure iSCC in detail. The proposed method consists of two steps: information retrieval and information-incorporated analysis. We will then discuss some practical considerations when implementing the proposed method.

### 2.1 iSCC

iSCC procedure mainly includes two steps. Step I: information retrieval aims to find disease-related features by leveraging existing publications through text mining techniques and assigning appropriate feature weights for our objective function. Step II: information-incorporated analysis is the sparse convex clustering procedure based on the updated weights in Step I and transformed omics datasets.

**Step I: Information retrieval.** To identify disease-related features, we construct a search for the co-occurrence of each feature and disease of interest. Take PubMed as an example. We can specify a keyword search of “(PGR[Title/Abstract]) AND (breast cancer[Title/Abstract])” in the search field. This search allows us to count how frequently progesterone receptor (PGR) is mentioned together with breast cancer in the same study. Further refinement of the search field is available based on the purpose of the study. More detailed information is available at https://pubmed.ncbi.nlm.nih.gov/help/#search-tags. For illustration, we focus on publications with a pair of a feature and a disease in their titles or abstracts.

Denote $f_{j}$ as the co-occurrence of feature j and a disease of interest, and F as the set of co-occurrences of all feature–disease pairs in a dataset such that $f_{j}\in \;F$. We assume that for a given feature–disease pair, the more publications got identified, the more likely the feature is to be an informative feature related to the mechanism of the disease. Since we aim to select informative features, we enforce different weights based on their co-occurrence with the disease of interest, putting less penalty on more informative features. Specifically, we define the weight $z_{j}$ for the feature j as: where $f_{T}$ is a user-specified threshold of the co-occurrence of a feature–disease pair in the set F. Researchers can define $f_{T}$ based on their knowledge. We suggest using $f_{T}=f_{0.9}$, the 90th percentile of the co-occurrence in the set F since the co-occurrence distribution is highly skewed and only a few features might be informative. Text mining is a fast-growing research area, and many text mining tools can serve our purpose here, such as MedMiner, PubMatrix, and easyPubMed. To illustrate our proposed procedure, we use easyPubMed to mine publications deposited in PubMed in this study. Compared to web-based text mining tools, easyPubMed as an R package does not require maintenance from the developers. Thus, it is more convenient to implement.


(1) zj=0.5, if fj>fT 1, otherwise

**Step II: Information-incorporated analysis.** Before integrating multiple omics datasets, we need a standardization procedure. We perform a feature-level centering and scaling each omics dataset by its standard deviation. Then we concatenate all transformed omics datasets into one big matrix $X\in R^{n\times p}$ as our input data, where n is the number of samples and p is the total number of features. Let $X_{i\cdot },\;i=1,\ldots ,n$, be the ith sample in the concatenated dataset and rewrite X in a feature-level way, $X=\left(x_{1},\ldots ,x_{p}\right)$ where $x_{j}$ is a column vector for the jth feature. Similar to sparse convex clustering (Wang *et al.* 2018), we can express the objective function of iSCC with the updated feature weight $z_{j}$ from step I as: where U is the cluster center attached to the concatenated matrix X, $u_{j}$ is the cluster center for the jth feature, $U_{i_{1}\cdot }$ is the cluster center for the $i_{1}$th sample, $E=\left\{l=\left(i_{1},i_{2}\right):\;1\leq i_{1}<i_{2}\leq n\right\}$, and $v_{l}=U_{i_{1}\cdot }-U_{i_{2}\cdot }$ represents the difference between two cluster centers. Tuning parameters $\gamma_{1}$ and $\gamma_{2}$ control the number of clusters and the number of informative features, respectively. The non-negative sample weight $w_{l}=w_{i_{1},i_{2}}=\iota_{\left\{i_{1},i_{2}\right\}}^{d}\exp\left(-\phi {\left\|X_{i_{1}\cdot }-X_{i_{2}\cdot }\right\|}_{2}^{2}\right)$ where $\iota_{\left\{i_{1},i_{2}\right\}}^{d}=1$ if $i_{2}$ is among $i_{1}\prime s$ d-nearest-neighbors or vice versa and 0 otherwise, ϕ=0.5, and ${\left\|\cdot \right\|}_{2}$ is the L_2_-norm. The second term of (2) is related to the fused lasso penalty (Tibshirani *et al.* 2005), which encourages samples’ centers to be fused with similar rows. The feature-weight $z_{j}$ in the third group-lasso term of (2) plays an important role in selecting features. We implement the alternating minimization algorithm (AMA) (Tseng 1991) to solve our objective function, which is computationally efficient.


(2) $$\begin{array}{c} \underset{U\in R^{n\times p}}{\min}\frac{1}{2}\sum_{j=1}^{p}{\left\|x_{j}-u_{j}\right\|}_{2}^{2}+\;\gamma_{1}\sum_{l\in E}w_{l}{\left\|v_{l}\right\|}_{2}+\gamma_{2}\sum_{j=1}^{p}z_{j}{\left\|u_{j}\right\|}_{2},\\ s.t.\;U_{i_{1}\cdot }-U_{i_{2}\cdot }-v_{l}=0 \end{array}$$

### 2.2 Practical considerations

When applying iSCC, the sample weight $w_{i_{1},i_{2}}$ and the feature weight $z_{j}$ are user-defined inputs, while $\gamma_{1}$ and $\gamma_{2}$ are tuning parameters. We specify feature weight $z_{j}$ through a text mining approach, as illustrated in Step I. For sample weight $w_{i_{1},i_{2}}$, we can update the sample weight as $w_{i_{1},i_{2}}=\iota_{\left\{i_{1},i_{2}\right\}}^{d}\exp\left(-\phi {\left\|{z\prime }^{T}\left(X_{i_{1}\cdot }-X_{i_{2}\cdot }\right)\right\|}_{2}^{2}\right)$, where z′ is a vector such that $z_{j}^{\prime }=1\;if\;z_{j}=0.5$ and $z_{j}^{\prime }=0.5\;if\;z_{j}=1$ to emphasize the importance of informative features when calculating sample-wise distance. Two tuning parameters are included in the objective function of iSCC: $\gamma_{1}$ controls the number of clusters; $\gamma_{2}$ controls the number of selected features. We choose the information-based approach (Tan and Witten 2015, Wang and Allen 2021) to select tunings instead of the stability-based approach (Wang 2010, Fang and Wang 2012), which is often computationally intensive.

When the numbers of clusters and informative features are known, we can first fix $\gamma_{2}=1\;$and fit iSCC with a sequence of $\gamma_{1}$ to get the $\gamma_{1}$ that gives the closest number of clusters to the known number. We then fit iSCC with the chosen $\gamma_{1}$ and a sequence of $\gamma_{2}$ to get the $\gamma_{2}$ that gives the closest number of features. We repeat this procedure until $\gamma_{1}$ and $\gamma_{2}$ stabilized. When the numbers of clusters and features are unknown, we adopt the Bayesian information criterion (BIC) based approach to choose $\gamma_{1}$ and $\gamma_{2}$ (Tan and Witten 2015, Wang and Allen 2021). We first fit iSCC with a fixed $\gamma_{2}=1\;$and a sequence of $\gamma_{1}$. Then we choose the $\gamma_{1}$ that minimizes $\mathrm{BI}C_{\mathrm{cluster}}=\frac{\mathrm{RS}S_{\mathrm{act}}}{\pi_{\mathrm{act}}}+n_{\mathrm{clust}}\times \log\left(n\times p_{\mathrm{act}}\right)$, where $\mathrm{RS}S_{\mathrm{act}}=\;{\left\|X_{\mathrm{act}}-{\hat{U}}_{\mathrm{act}}\right\|}_{2}^{2},\;\pi_{\mathrm{act}}=\frac{1}{n}{\left\|X_{\mathrm{act}}-{\bar{X}}_{\mathrm{act}}\right\|}_{2}^{2},\;\mathrm{act}$ represents the dataset including selected features only, $n_{\mathrm{clust}}$ is the number of identified clusters, and $p_{\mathrm{act}}$ is the number of selected features. We then fit iSCC with the chosen $\gamma_{1}$ and a sequence of $\gamma_{2}$ to get the $\gamma_{2}$ that gives the minimum of $\mathrm{BI}C_{\mathrm{feature}}=\sum_{j=1}^{p}\frac{\mathrm{RS}S_{j}}{\pi_{j}}+n_{\mathrm{clust}}\times p_{\mathrm{act}}\times \log\left(n\right)$, where $\mathrm{RS}S_{j}={\left\|X_{\cdot j}-{\hat{U}}_{\cdot j}\right\|}_{2}^{2},\pi_{j}=\frac{1}{n}{\left\|X_{\cdot j}-{\bar{X}}_{\cdot j}\right\|}_{2}^{2}$. Then repeat the procedure until $\gamma_{1}$ and $\gamma_{2}$ stabilized. When either the number of clusters or the number of features is unknown, the tuning procedure is similar to the above, except that choosing $\gamma_{1}$ that minimizes $\mathrm{BI}C_{\mathrm{cluster}}$ and $\gamma_{2}$ that gives the closest number of features or choosing $\gamma_{1}$ that gives the closest number of clusters and $\gamma_{2}$ that minimizes $\mathrm{BI}C_{\mathrm{feature}}$. In summary, $\mathrm{BI}C_{\mathrm{cluster}}$ is used to select the number of clusters based on selected features with a degree of freedom of $n_{\mathrm{clust}}$. $\mathrm{BI}C_{\mathrm{feature}}$ is then used to select the number of features with the estimated number of clusters from the previous step and the degree of freedom of this step is $n_{\mathrm{clust}}\times p_{\mathrm{act}}$.

## 3 Results

### 3.1 Simulation studies

We conduct simulation studies to evaluate the performance of our proposed method, iSCC, in comparison with SCC, K-means, Sparse K-means (Sun *et al.* 2012, Witten and Tibshirani 2010), iCluster+ (Mo *et al.* 2013), and Bayesian Consensus Clustering (BCC) (Lock and Dunson 2013) with available R packages (*scvxlustr*, *sparcl*, *iClusterPlus*) and code. We implement the adjusted Rand index (ARI) (Hubert and Arabie 1985) to measure the accuracy of clustering results. The ARI calculates the similarity between the estimated clustering assignment and the actual group label. It ranges from –1 to 1, and a larger value implies a better clustering result. We report the false negative rate (FNR), the false positive rate (FPR), and the Matthews correlation coefficient (MCC) (Matthews 1975) to evaluate the performance of feature selection for each method. We assume that the number of clusters and features are known for fair comparison between methods.

**S1 spherical setting**: The numbers of features are $p_{1}=100$ and $p_{2}=200\;$for two datasets $X^{1}$ and $X^{2}$, respectively. For $X^{1}$, the first 30 informative features are generated from a multivariate normal distribution $\mathrm{MVN}(\mu_{k},\sigma^{2}\times I_{30})$ where $\mu_{1}={\left(3\times 1_{10}^{T},0_{20}^{T}\right)}^{T}$, $\mu_{2}={\left(0_{10}^{T},2\times {1_{10}^{T},\;0}_{10}^{T}\right)}^{T}$, $\mu_{3}={\left(0_{20}^{T},1_{10}^{}\right)}^{T}$, and σ=0.5. The fourth cluster is from the background distribution [i.e. all features are from $N(0,\sigma^{2})$]. In addition, we generate a noisy clustering pattern using features 31–50 from $\mathrm{MVN}(\mu_{k}^{\prime },\sigma^{2}{\times I}_{20})$ where $\mu_{1}^{\prime }={\left(2\times 1_{10}^{T},0_{10}^{T}\right)}^{T}$, $\mu_{2}^{\prime }={\left(0_{10}^{T},\;1_{10}^{T}\right)}^{T}$. The remaining noninformative features are generated from $N(0,\sigma^{2})$. Similar setups are applied to $X^{2}$ except $\mu_{1}={\left(4\times 1_{10}^{T},0_{20}^{T}\right)}^{T}$, $\mu_{2}={\left(0_{10}^{T},3\times {1_{10}^{T},\;0}_{10}^{T}\right)}^{T}$, $\mu_{3}={\left(0_{20}^{T},2\times 1_{10}^{T}\right)}^{T}$, σ=1, and $\mu_{1}^{\prime }={\left(3\times 1_{10}^{T},0_{10}^{T}\right)}^{T}$, $\mu_{2}^{\prime }={\left(0_{10}^{T},2\times 1_{10}^{T}\right)}^{T}$.

**S2 spherical setting**: The number of features is $p_{1}=200$ and $p_{2}=500\;$for two datasets $X^{1}$ and $X^{2}$, respectively. Like S1, but the means of features are in different directions. The first 30 informative features of $X^{1}$ have $\mu_{1}={\left(3\times 1_{10}^{T},0_{20}^{T}\right)}^{T}$, $\mu_{2}={\left(0_{10}^{T},-2\times {1_{10}^{T},\;0}_{10}^{T}\right)}^{T}$, $\mu_{3}={\left(0_{20}^{T},\;1_{10}^{T}\right)}^{T}$, σ=0.5. Noisy features have $\mu_{1}^{\prime }={\left(2\times 1_{10}^{T},0_{10}^{T}\right)}^{T}$, $\mu_{2}^{\prime }={\left(0_{10}^{T},\;{-1}_{10}^{T}\right)}^{T}$. For $X^{2}$, $\mu_{1}={\left(4\times 1_{10}^{T},0_{20}^{T}\right)}^{T}$, $\mu_{2}={\left(0_{10}^{T},-3\times {1_{10}^{T},\;0}_{10}^{T}\right)}^{T}$, $\mu_{3}={\left(0_{20}^{T},2\times 1_{10}^{T}\right)}^{T}$, σ=1, and $\mu_{1}^{\prime }={\left(3\times 1_{10}^{T},0_{10}^{T}\right)}^{T}$, $\mu_{2}^{\prime }={\left(0_{10}^{T},-2\times 1_{10}^{T}\right)}^{T}.$

**S3 nonspherical setting with two half-moons**: The number of features is $p_{1}=100$ and $p_{2}=200\;$for two datasets $X^{1}$ and $X^{2}$, respectively. Each pair of the first ten informative features in either $X^{1}$ or $X^{2}$ makes up two interlocking half-moons through a combination of cosine and sine functions. Again, a noisy clustering pattern is generated using the second 10 features (noisy features) from $\mathrm{MVN}(\mu_{k}^{\prime },I_{10})$ where $\mu_{1}^{\prime }={\left(2\times 1_{5}^{T},0_{5}^{T}\right)}^{T}$, $\mu_{2}^{\prime }={\left(0_{5}^{T},\;1_{5}^{T}\right)}^{T}$. The remaining noninformative features are generated from N(0,1). Supplementary Figure S2 reveals one example of two interlocking half-moons using one pair of informative features.

For each setting, we evaluate the effect of the accuracy rate of informative feature weights ($\theta_{i}=1,0.7,0.5,0.3$) and the accuracy rate of noninformative feature weights ($\theta_{ni}=0.9,\;0.8,0.7$) individually while assuming a perfect accuracy rate for another feature set. Then we also consider their joint effects with varying accuracy rates ($\theta_{\mathrm{both}}=0.9,\;0.8,0.7$) simultaneously. More specifically, $\theta_{\mathrm{both}}$ is defined as the proportion of both informative and noninformative features with correct weights. All the results are based on 100 replicates.

#### 3.1.1 Simulation results

The simulation results are summarized in Table 1. The proposed method, iSCC, performs the best in terms of clustering accuracy with the highest ARI and feature selection with the highest MCC in all three settings when proper weights are assigned to both informative and noninformative feature based on prior information ($\theta_{i}=1$). The means of ARI are 0.91, 0.92, and 0.91, and the means of MCC are 0.96, 0.96, and 1.00 in three settings for iSCC when $\theta_{i}=1$, respectively. In addition, iSCC outperforms most other methods, including SCC, K-means, iCluster+, and BCC, for both clustering accuracy and feature selection in all ten scenarios with different accuracy rates of feature weights in both **S1** and **S2**. In **S1**, the lowest means of ARI (0.88) and MCC (0.59) among all iSCC occur with $\theta_{\mathrm{both}}=0.7$, which is still higher than most compared methods except for Sparse K-means, which has ARI = 0.89 and MCC = 0.67. Similarly, in **S2**, iSCC with $\theta_{\mathrm{both}}=0.7$ has the lowest ARI (0.78) and MCC (0.67) among all iSCC, which are still higher than most compared methods except for Sparse K-means with ARI = 0.87 and MCC = 0.71. When $\theta_{i}>0.5$, or $\theta_{\mathrm{both}}>0.8$, iSCC performs better than the best performer, Sparse K-means, among compared methods regarding clustering accuracy in **S3**. In terms of feature selection, iSCC with $\theta_{i}>0.5$ or $\theta_{ni}>0.8$ performs better or comparable with the best performer, iCluster+, among compared methods in **S3**. The clustering accuracy and the performance of feature selection for iSCC generally decrease when the accuracy rate of feature weights decreases. We only compare feature selection among iSCC, SCC, Sparse K-means, and iCluster+ since K-means and BCC cannot select features.

**Table 1.** Simulation results are based on 100 replicates, including mean and standard deviation (SD) of the adjusted Rand index (ARI), false negative rate (FNR), false positive rate (FPR), and Matthews correlation coefficient (MCC).^a^^,^^b^

Method	ARI (mean [SD])	FNR (mean [SD])	FPR (mean [SD])	MCC (mean [SD])
S1 spherical setting				
iSCC ($\theta_{i}=1$)	**0.91 (0.01)**	0.04 (0.02)	0.01 (0.01)	**0.96 (0.02)**
iSCC ($\theta_{i}=0.7$)	0.90 (0.04)	0.18 (0.04)	0.05 (0.02)	0.77 (0.05)
iSCC ($\theta_{i}=0.5$)	0.90 (0.04)	0.26 (0.04)	0.07 (0.01)	0.67 (0.04)
iSCC ($\theta_{i}=0.3$)	0.88 (0.07)	0.30 (0.04)	0.08 (0.01)	0.61 (0.03)
iSCC ($\theta_{ni}=0.9$)	0.90 (0.05)	0.10 (0.03)	0.04 (0.02)	0.85 (0.04)
iSCC ($\theta_{ni}=0.8$)	**0.91 (0.02)**	0.21 (0.08)	0.04 (0.02)	0.78 (0.04)
iSCC ($\theta_{ni}=0.7$)	0.90 (0.04)	0.26 (0.07)	0.03 (0.01)	0.75 (0.04)
iSCC ($\theta_{\mathrm{both}}=0.9$)	**0.91 (0.03)**	0.15 (0.04)	0.04 (0.01)	0.80 (0.04)
iSCC ($\theta_{\mathrm{both}}=0.8$)	0.89 (0.05)	0.22 (0.05)	0.08 (0.02)	0.69 (0.06)
iSCC ($\theta_{\mathrm{both}}=0.7$)	0.88 (0.05)	0.30 (0.06)	0.10 (0.04)	0.59 (0.08)
SCC	0.74 (0.06)	0.33 (0.01)	0.09 (0.01)	0.58 (0.01)
K-means	0.74 (0.20)	N.A.	N.A.	N.A.
Sparse K-means	0.89 (0.14)	0.24 (0.07)	0.08 (0.01)	0.67 (0.06)
iCluster+	0.77 (0.10)	0.38 (0.05)	0.10 (0.01)	0.52 (0.07)
BCC	0.64 (0.18)	N.A.	N.A.	N.A.
S2 spherical setting				
iSCC ($\theta_{i}=1$)	**0.92 (0.02)**	0.02 (0.02)	0.01 (<0.01)	**0.96 (0.02)**
iSCC ($\theta_{i}=0.7$)	0.89 (0.08)	0.18 (0.03)	0.02 (0.01)	0.80 (0.05)
iSCC ($\theta_{i}=0.5$)	0.88 (0.09)	0.26 (0.04)	0.03 (<0.01)	0.71 (0.03)
iSCC ($\theta_{i}=0.3$)	0.83 (0.12)	0.30 (0.04)	0.03 (<0.01)	0.67 (0.03)
iSCC ($\theta_{ni}=0.9$)	0.90 (0.08)	0.13 (0.07)	0.01 (0.00)	0.88 (0.03)
iSCC ($\theta_{ni}=0.8$)	0.86 (0.11)	0.18 (0.10)	0.01 (0.01)	0.83 (0.05)
iSCC ($\theta_{ni}=0.7$)	0.82 (0.13)	0.24 (0.09)	0.01 (0.01)	0.78 (0.05)
iSCC ($\theta_{\mathrm{both}}=0.9$)	0.89 (0.10)	0.20 (0.08)	0.01 (0.01)	0.84 (0.04)
iSCC ($\theta_{\mathrm{both}}=0.8$)	0.82 (0.13)	0.25 (0.10)	0.02 (0.01)	0.76 (0.05)
iSCC ($\theta_{\mathrm{both}}=0.7$)	0.78 (0.13)	0.34 (0.12)	0.02 (0.01)	0.67 (0.06)
SCC	0.65 (0.07)	0.33 (0.01)	0.03 (<0.01)	0.63 (0.01)
K-means	0.77 (0.20)	N.A.	N.A.	N.A.
Sparse K-means	0.87 (0.14)	0.24 (0.08)	0.03 (0.00)	0.71 (0.06)
iCluster+	0.77 (0.11)	0.59 (0.04)	0.02 (<0.01)	0.51 (0.06)
BCC	0.64 (0.19)	N.A.	N.A.	N.A.
S3 nonspherical setting with two half-moons				
iSCC ($\theta_{i}=1$)	**0.91 (0.12)**	0.00 (0.00)	0.00 (0.00)	**1.00 (0.00)**
iSCC ($\theta_{i}=0.7$)	0.82 (0.22)	0.13 (0.07)	0.01 (0.01)	0.88 (0.07)
iSCC ($\theta_{i}=0.5$)	0.66 (0.37)	0.25 (0.08)	0.02 (0.01)	0.75 (0.09)
iSCC ($\theta_{i}=0.3$)	0.49 (0.43)	0.28 (0.10)	0.01 (0.01)	0.73 (0.09)
iSCC ($\theta_{ni}=0.9$)	0.63 (0.34)	0.09 (0.12)	0.08 (0.23)	0.80 (0.23)
iSCC ($\theta_{ni}=0.8$)	0.54 (0.38)	0.07 (0.14)	0.23 (0.35)	0.62 (0.24)
iSCC ($\theta_{ni}=0.7$)	0.44 (0.38)	0.03 (0.09)	0.33 (0.35)	0.48 (0.22)
iSCC ($\theta_{\mathrm{both}}=0.9$)	0.68 (0.33)	0.10 (0.09)	0.14 (0.29)	0.70 (0.26)
iSCC ($\theta_{\mathrm{both}}=0.8$)	0.48 (0.38)	0.15 (0.14)	0.33 (0.38)	0.46 (0.24)
iSCC ($\theta_{\mathrm{both}}=0.7$)	0.40 (0.33)	0.15 (0.18)	0.45 (0.36)	0.30 (0.15)
SCC	0.41 (0.35)	0.02 (0.09)	0.24 (0.11)	0.44 (0.14)
K-means	0.47 (0.07)	N.A.	N.A.	N.A.
Sparse K-means	0.67 (0.03)	0.00 (0.00)	0.10 (0.06)	0.64 (0.12)
iCluster+	0.45 (0.25)	0.00 (0.00)	0.04 (0.00)	0.80 (0.00)
BCC	0.55 (0.09)	N.A.	N.A.	N.A.

a $\theta_{i},\;\theta_{ni},\theta_{\mathrm{both}}$ are the accuracy rates of informative feature weights, noninformative feature weights, and both informative and noninformative feature weights, respectively. Three simulation settings are considered. S1 spherical setting: $K=4,\;n=80,\;p_{1}=100,\;p_{2}=200$; S2 spherical setting: $K=4,\;n=80,\;p_{1}=200,\;p_{2}=500$; S3 nonspherical setting with two half-moons: $K=2,\;n=80,\;p_{1}=100,\;p_{2}=200$. All clusters within each setting have an equal sample size. The best ARI and MCC in each setting are in bold.

b Since K-means and BCC cannot select featues, there is no FNR, FPR, or MCC for them. N.A. stands for not applicable.

The simulation results when the clusters with imbalanced sample size are summarized in Supplementary Table S1. iSCC outperforms all other methods, including SCC, K-means, Sparse K-means, iCluster+, and BCC, for clustering accuracy in all ten scenarios with different accuracy rates of feature weights in both **S1** and **S2**. In terms of feature selection, iSCC with $\theta_{i}>0.5,\;\theta_{ni}\geq 0.7$ or $\theta_{\mathrm{both}}>0.7$ performs better than the best performer, SCC, among compared methods in **S1** and **S2**. In **S3**, iSCC outperforms other methods when $\theta_{i}>0.7,\;\theta_{ni}>0.8\;$or $\theta_{\mathrm{both}}>0.7$ regarding clustering accuracy and performs better than other methods when $\theta_{i}>0.3$ regarding feature selection.

### 3.2 Real data applications

In this section, we apply our method, iSCC, to several multi-omics data from the Cancer Genome Atlas (TCGA) breast cancer and mRNA expression data from human lung cancer. We evaluate the performance of our method against other clustering methods, including SCC, K-means, Sparse K-means, iCluster+, and BCC. We use the above-mentioned BIC-based approach to tune hyperparameters for iSCC and SCC. The gap statistic (Tibshirani *et al.* 2001) is implemented to select the number of clusters for K-means, Sparse K-means, iCluster+, and BCC. In addition, we perform follow-up analysis on clinical variables and investigate the biological interpretation for the identified clusters and features.

#### 3.2.1 TCGA breast cancer multi-omics data

Breast cancer is a well-known heterogeneous disease that has been extensively studied. The TCGA, a landmark cancer genomics program, compiles a large amount of genomic, epigenomic, transcriptomic, and proteomic data spanning 33 different cancer types for public use. We utilize the TCGA breast cancer data (Cancer Genome Atlas Network 2012) and preprocess the data as suggested by Lock and Dunson (Lock and Dunson 2013). In total, we have 348 tumor samples with RNA gene expression data for 645 genes, DNA methylation data for 574 probes, miRNA expression data for 423 miRNAs, and reverse phase protein array data for 171 proteins. PAM50 is a gene-based classifier that classifies breast cancer into five molecular intrinsic subtypes: Luminal A, Luminal B, HER2-enriched, Basal-like, and Normal-like with distinct biological properties and prognoses using 50 genes (Parker *et al.* 2009). Since there are no true labels, we use the PAM50 as a reference to see if our identified clusters are biologically meaningful. We merge subtypes luminal A and luminal B into one luminal-like subtype since they are often regarded as one big group (Choi *et al.* 2014, Wang and Allen 2021).

Feature-wise centering and data-wise scaling are performed before applying our method. The distributions of data from different platforms before and after transformation are shown in Supplementary Fig. S3a and b, respectively. All datasets appear to be symmetric after transformation. Through easyPubMed, the frequency of co-occurrence between a feature and breast cancer on PubMed ranges from 0 to 4201 for gene expression data; 0 to 1 for methylation data; 0 to 450 for miRNA data; and 0 to 22 053 for protein data. Supplementary Figure S4 shows the histograms of co-occurrence for data from different platforms for TCGA breast cancer.

**Figure 1.**
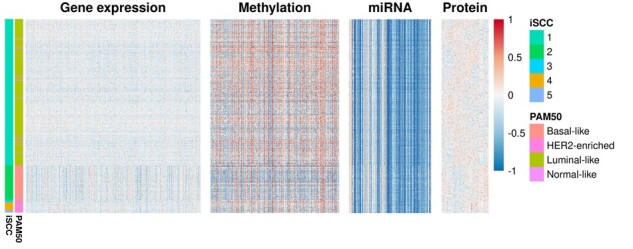
Heatmap for different platforms (gene expression, methylation, miRNA, and protein data) of TCGA breast cancer. Rows (i.e. samples) are ordered by the iSCC cluster assignments. Left bars include iSCC assignments and PAM50 assignments.

Our method outperforms other methods with an ARI of 0.8 compared to alternative approaches with an ARI ranging from 0.32 to 0.77 (Table 2). We present the matching matrix comparing iSCC-identified clusters after removing clusters with less than three samples with the PAM50 subtypes in Table 3. Table 3 also shows different proportions of samples with the expression of clinical variables and 5-year survival rates for the iSCC clusters. Cluster 1 from iSCC mainly comprises subjects belonging to the luminal-like subtype, characterized by the expression of estrogen receptor (ER) and/or progesterone receptor (PR). We observe consistent patterns that more than 80% of patients in iSCC cluster 1 have ER+ and/or PR+. iSCC cluster 2 is well aligned with the basal-like subtype, also known as ER-, PR-, HER2- triple-negative breast cancer, with a poor prognosis. Cluster 2 has the lowest five-year survival rate among all other identified clusters. iSCC clusters 3, 4, and 5 are closely related to the HER2-enriched subtype. More than half of patients in these clusters have HER2 expression. In addition, iSCC reports 31 selected features (Supplementary Table S2). At least one breast cancer-related publication has reported each of the selected features, even though only 29 selected features were originally receiving informative feature weights. *PGR* and *ESR1*, coding genes for PR and ER respectively, in the gene expression data and PR in the protein data, are selected as expected. More than 1000 publications mention them together with breast cancer. Another selected gene, *FOXA1*, is a hallmark of ER+ breast cancer and influences therapeutic responses in breast cancer (Arruabarrena-Aristorena *et al.* 2020). Several well-studied miRNAs have also been identified. For example, miR-155 has been closely related to breast cancer progression and drug resistance (Mattiske *et al.* 2012, Yu *et al.* 2015). miR-205 is differentially expressed in the different subtypes of breast cancer (Plantamura *et al.* 2020). Figure 1 shows the heatmap of TCGA multi-omics data for breast cancer together with the iSCC-generated clustering assignments and PAM50 labels. iSCC clusters are closely related to PAM50 subtypes with different patterns across four platforms of omics data.

**Table 2.** Adjusted Rand index (ARI) of different methods for TCGA breast cancer multi-omics dataset and human lung cancer mRNA dataset.

	TCGA breast cancer multi-omics data	Human lung cancer mRNA data
Method	ARI	ARI

iSCC	0.80	0.78
SCC	0.77	0.74
K-means	0.37	0.72
Sparse K-means	0.74	0.75
iCluster+	0.35	0.75
BCC	0.32	0.64

**Table 3.** The matching matrix between iSCC-identified clusters and PAM50 subtypes, summary data of iSCC-identified clusters based on clinical variables for the TCGA breast cancer multi-omics dataset.^a^

iSCC cluster	PAM50 subtype				Clinical variables			
	Basal-like	HER2-enriched	Luminal-like	Normal-like	ER+	PR+	HER2+	5-Year survival (SE)
1	0	11	227	2	96%	83%	11%	0.85 (0.04)
2	57	0	0	0	9%	5%	0%	0.71 (0.12)
3	0	4	0	0	50%	25%	50%	0.75 (0.22)
4	0	12	1	0	46%	15%	92%	1.00 (0.00)
5	0	4	0	0	0%	0%	75%	1.00 (0.00)

a ER+ means breast cancers with estrogen receptors; PR+ means breast cancer with progesterone receptors; HER2+ means breast cancers with higher-than-normal levels of HER2 protein; 5-year survival (se) means the 5-year survival probabilities using the Kaplan–Meier estimator with standard error.

#### 3.2.2 Human lung cancer mRNA data

Lung cancer subtyping according to a molecular basis could help predict a patient’s treatment outcome and develop targeted therapy. We use the mRNA gene expression data based on a cohort of people with or without lung cancer (Bhattacharjee *et al.* 2001). We select the top 500 out of 12 625 genes with the largest expression variance for 56 samples. There are four groups, including pulmonary carcinoid (Carcinoid: *n* = 20), colon cancer metastasis (Colon: *n* = 13), small cell carcinoma (Small cell: *n* = 6), and normal lung (Normal: *n* = 17).

The data distribution is available in Supplementary Fig. S5, which is close to symmetric. Since the gene expression was assayed using the Affymetrix 95av2 GeneChip brand oligonucleotide array. We first convert the array probe name into gene name using https://biit.cs.ut.ee/gprofiler/convert. Then through easyPubMed, the co-occurrence between a gene and lung cancer on PubMed ranges from 0 to 505. Supplementary Figure S6 shows the histogram of co-occurrence for mRNA data for human lung cancer.

**Figure 2.**
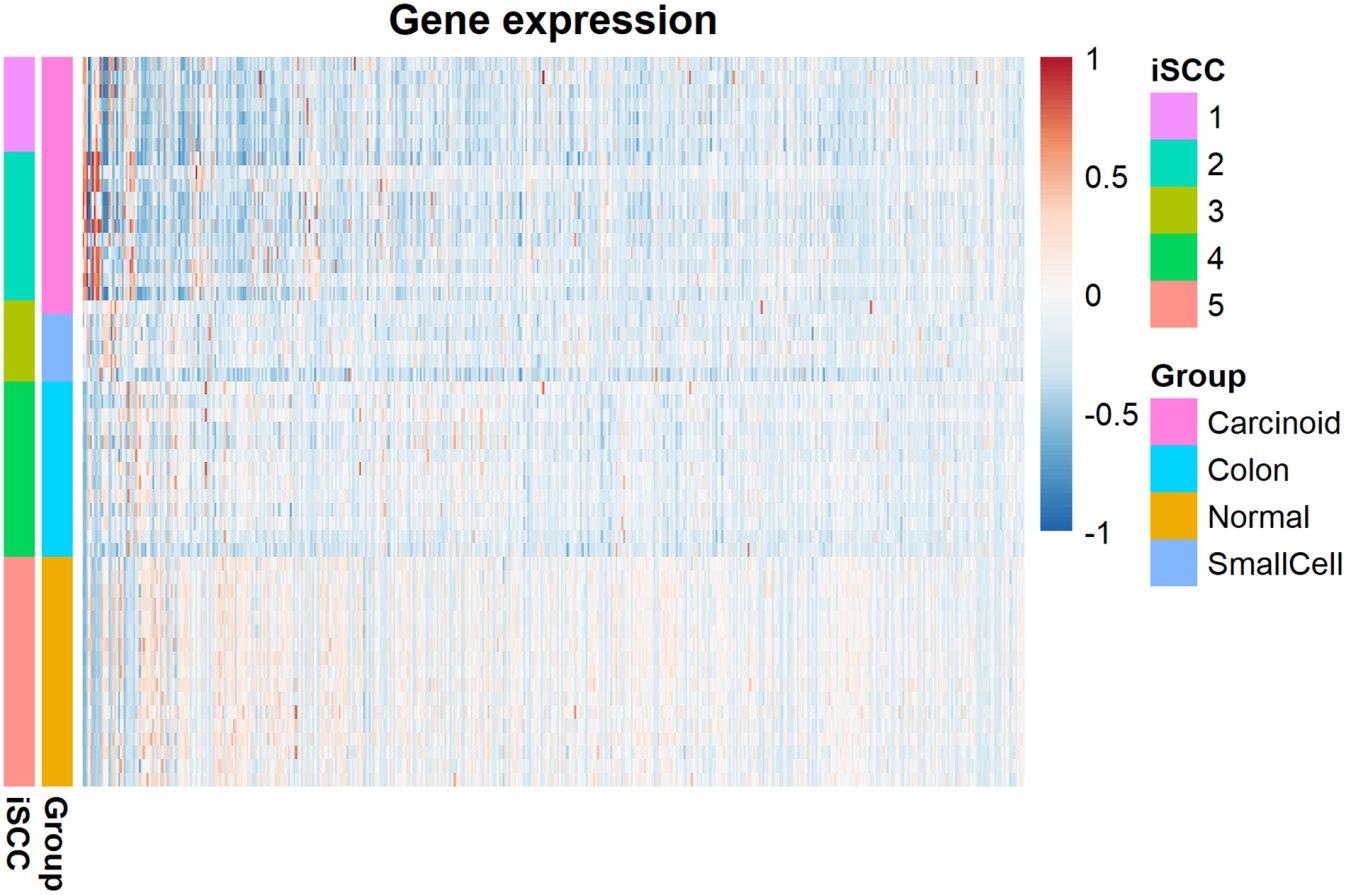
Heatmap for gene expression data of human lung cancer. Rows (i.e. samples) are ordered by the iSCC cluster assignments. Left bars include iSCC assignments and the known groups.

Our method maintains the highest ARI score of 0.78 compared to others (Table 2). Supplementary Table S2 presents iSCC-selected features, and Table 4 tabulates the matching matrix between iSCC-identified clusters (after removing clusters with less than three samples) and four known groups. Clusters 1 and 2 from iSCC are samples from the Carcinoid group. They could be two subtypes of Carcinoid. Means of mRNA expression and *P*-values of *t*-test between iSCC-identified clusters 1 and 2 for the 18 selected genes are shown in Supplementary Table S3. *TTR*, *ASCL1*, *ALDH1A1*, and *NKX2-1* are significantly differentially expressed between clusters 1 and 2 after Bonferroni adjustment for multiple testing (significant threshold = 0.0028). Many literatures have reported that these differentially expressed genes are related to the mechanism and subtypes of lung cancer (Roudi *et al.* 2015, Hao *et al.* 2016, Lee *et al.* 2019, Baine *et al.* 2020, Pozo *et al.* 2021). iSCC clusters 4 and 5 match the Colon and the Normal groups separately. iSCC cluster 3 is closely related to the Small cell group. Among the 18 features selected by iSCC (Supplementary Table S2), most have been reported in at least one lung cancer-related publication. *MUC1*, for example, is associated with a poor prognosis of lung cancer (Lakshmanan *et al.* 2015). Pro-Gastrin-Releasing-Peptide (Pro-GRP), a precursor of Gastrin-Releasing-Peptide coded by gene *GRP*, has been used as a tumor marker for small-cell lung cancer (Cavalieri *et al.* 2018). *IL6* has recently been demonstrated to promote metastasis of non-small cell lung cancer (Liu *et al.* 2020). Figure 2 is the heatmap of gene expression data for lung cancer. As described above, iSCC clusters are well aligned with known groups, with a clear separation between clusters.

**Table 4.** The matching matrix between iSCC-identified clusters and four known groups for the human lung cancer mRNA dataset.

iSCC cluster	Group			
	Carcinoid	Colon	Small cell	Normal
1	7	0	0	0
2	11	0	0	0
3	1	0	5	0
4	0	13	0	0
5	0	0	0	17

## 4 Discussion

The clustering analysis for disease subtyping faces two critical challenges: a lack of ways to utilize existing information and incorrectly identifying disease-related clusters. We develop an information-incorporated sparse convex clustering procedure, iSCC, for disease subtyping and biomarker identification. Simulation studies show that the proposed method outperforms other clustering methods when the accuracy rate of feature weights is relatively high. In addition, the real data analysis of omics data demonstrates that iSCC can identify more accurate disease subtypes and select biologically meaningful biomarkers.

We incorporate prior information from published studies through text mining techniques to enhance the identification of clinically meaningful disease subtypes and disease-related biomarkers. In our study, the text mining procedure only includes omics data for demonstration purposes. However, the method itself can be applied to other types of features, clinical or nonclinical, as long as there are suitable data resources for text mining. We take a binary weighting scheme for different features based on their co-occurrence with the disease of interest during the information retrieval procedure. This weighting approach will be less affected by the large variation of $f_{j}$ and the publication bias through time that some omics data may have much more publications than others. Also, this weighting approach is easy to implement and more efficient for tuning. However, more complex feature weights, such as continuous weights, can be designed if needed. Continuous weighting schemes may give more information about each feature, but their tuning procedure could be more challenging. In addition, we observe highly skewed co-occurrence distribution, and we assume that only a few features might be informative as more cautious with text mining results. We understand that the searched results may contain noisy information when counting the feature frequency due to the nature of text mining. Therefore, we suggest a binary weighting using the 90th percentile of the co-occurrence of feature–disease pairs as the threshold to assign feature weights. Other thresholds can be applied if researchers observe different shapes of the co-occurrence distribution or believe that more features be informative based on prior knowledge.

The proposed method can offer alternative settings beyond the weighting scheme. In the information-incorporated analysis procedure, the second term of equation (2) encourages similar samples’ centers to be fused. For example, instead of the $L_{2}$-norm, researchers may also consider $L_{1}$- or $L_{\infty }$-norm, which results in a solution containing many zeros, forming a small set of clusters (Pelckmans *et al.* 2005). $L_{1}$-norm has an explicit solution as $L_{2}$-norm does, but $L_{\infty }$-norm does not and requires an efficient algorithm to solve it (Chi and Lange 2015). Compared to $L_{1}$- or $L_{\infty }$-norm, the use of $L_{2}$-norm is more computationally efficient (Pelckmans *et al.* 2005). Another consideration is parameter tuning, as selecting tuning parameters is not trivial. We choose the information-based approach with time expense in mind since computational efficiency is critical in multi-omics data analysis. However, other options, such as the stability-based approach (Wang 2010, Fang and Wang 2012), can be applied if computational cost is not of concern since it is computationally intensive due to the bootstrapping procedure.

While we have demonstrated the proposed method's superior performance compared to the alternative approach, several directions are still worth exploring in the future. First, the publications resulting from the information retrieval may not always be relevant to disease subtypes. Since text mining is rapidly evolving, more advanced tools are developing, potentially improving to retrieval of more robust and relevant information from unstructured data. Secondly, the current design of the proposed procedure may not benefit less studied diseases with few publications. Researchers may follow the same spirit as the proposed method but consider other data sources or prior knowledge based on experts for these diseases. Therefore, how to efficiently incorporate various types of information requires further investigation. Thirdly, our simulation studies only evaluate the impact of the spherical shape on noisy clustering patterns among spherical and nonspherical settings. It would be valuable to consider further the effect of different shapes of noisy clustering patterns under different setups for future work.

In conclusion, we introduce a clustering approach allowing prior information incorporation to leverage the existing and accumulated data resources. The proposed method responds to the need for disease subtyping and biomarker identification in precision medicine.

## Supplementary Material

btad417_Supplementary_Data

## Data Availability

The data underlying this article were derived from sources in the public domain: (1) the BCC package website at https://github.com/ttriche/bayesCC/tree/master/inst/extdata and (2) the online supplementary material of Bhattacharjee et al., 2001 [doi: https://10.1073/pnas.191502998]. The original access date was 10/2021.
