# Supplementary material for: Information-incorporated sparse convex clustering for disease subtyping
Source: Bioinformatics. 2023 Jun 29;39(7):btad417. doi: 10.1093/bioinformatics/btad417 (PMC10329496; doi:10.1093/bioinformatics/btad417)
Supplement: btad417_Supplementary_Data [file btad417_supplementary_data.docx]

**Information-incorporated sparse convex clustering for disease subtyping**

**Supplemental Materials**

**Supplemental Fig. 1.** Simulated dataset illustrating the existence of two types of clusters (disease-related clusters and noisy clusters). Assume that the three clusters defined by features 1-30 are disease-related, and the two clusters defined by features 31-50 are noisy clusters. It is challenging for most existing clustering methods to identify disease-related clusters only due to noisy clusters.


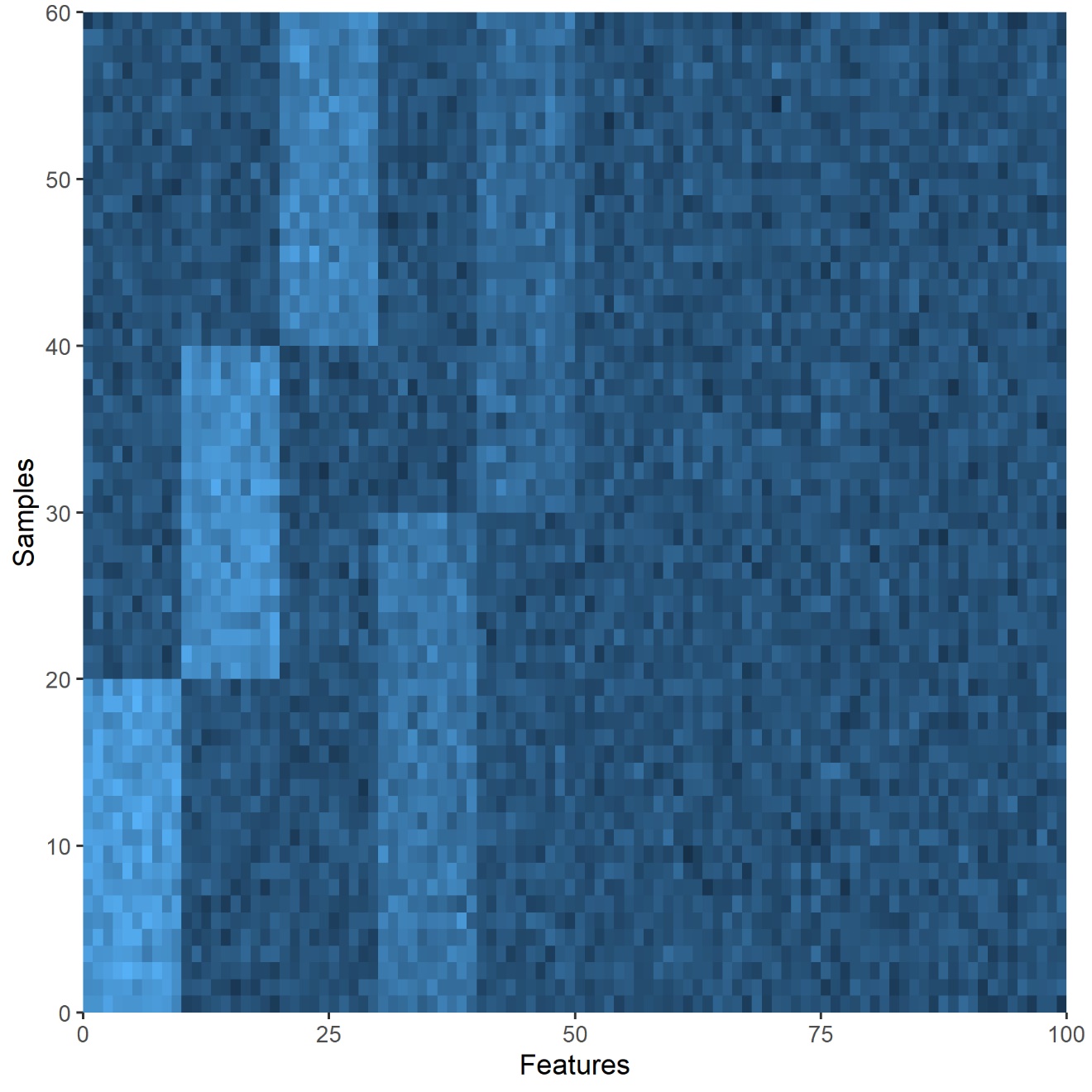


**Supplemental Fig. 2.** One example for the plot of two interlocking half-moons through one pair of informative features in the simulated dataset.

**
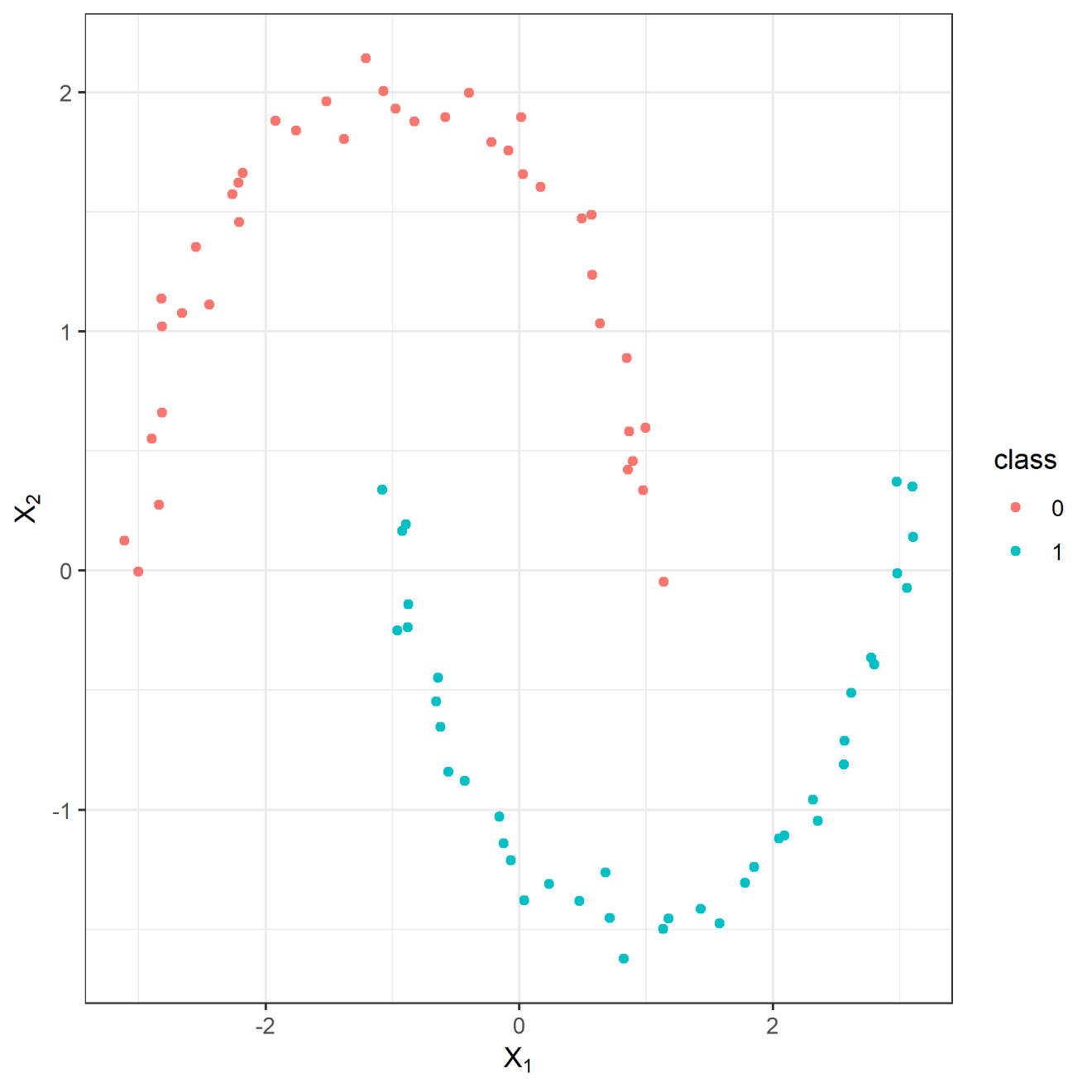
**

**Supplemental Fig. 3.** Histograms of gene expression data, methylation data, miRNA data, and protein data of TCGA breast cancer **(a)** before and **(b)** after transformation.

**(a)**


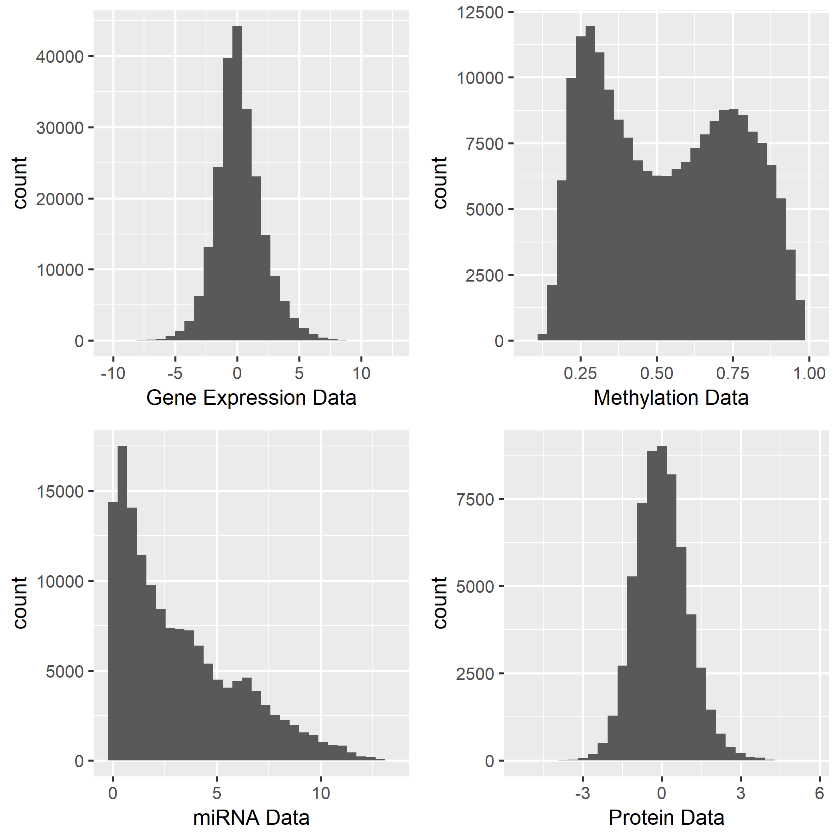


**(b)**


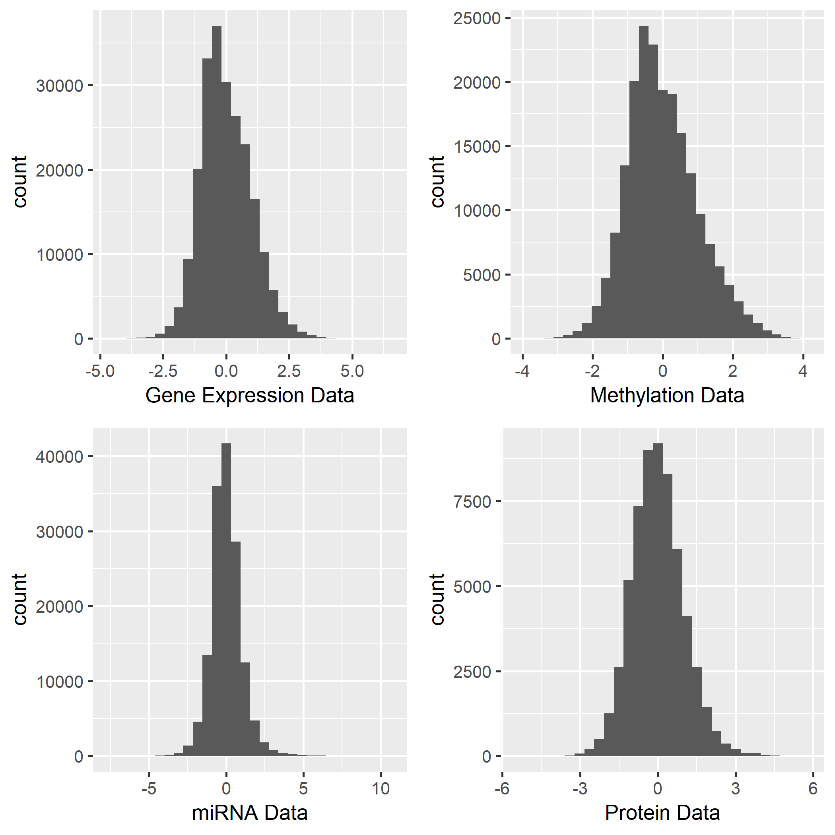


**Supplemental Fig. 4.** Histograms of co-occurrence between a feature and breast cancer on PubMed for different platforms (gene expression data, methylation data, miRNA data, and protein data) of TCGA breast cancer.


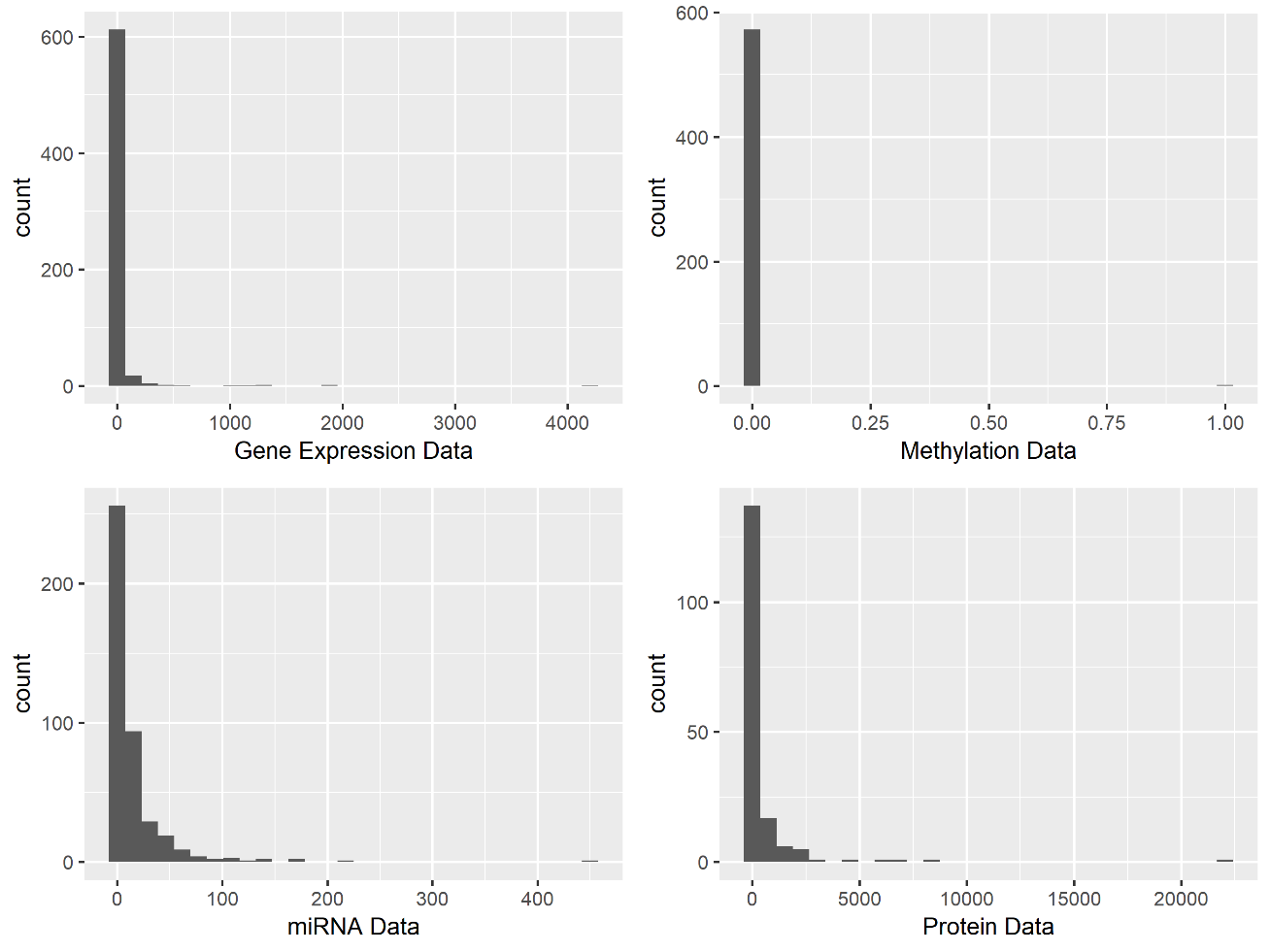


**Supplemental Fig. 5.** Histogram of gene expression data of human lung cancer


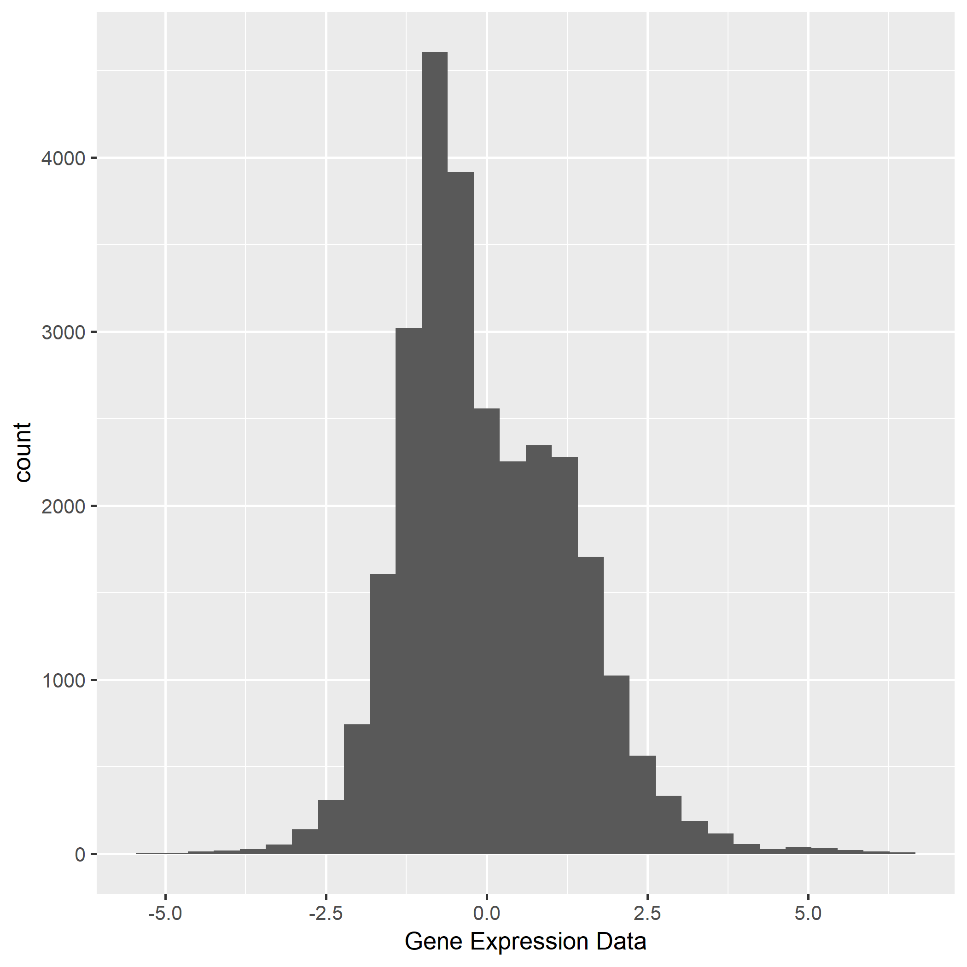


**Supplemental Fig. 6** Histogram of co-occurrence for mRNA data of human lung cancer


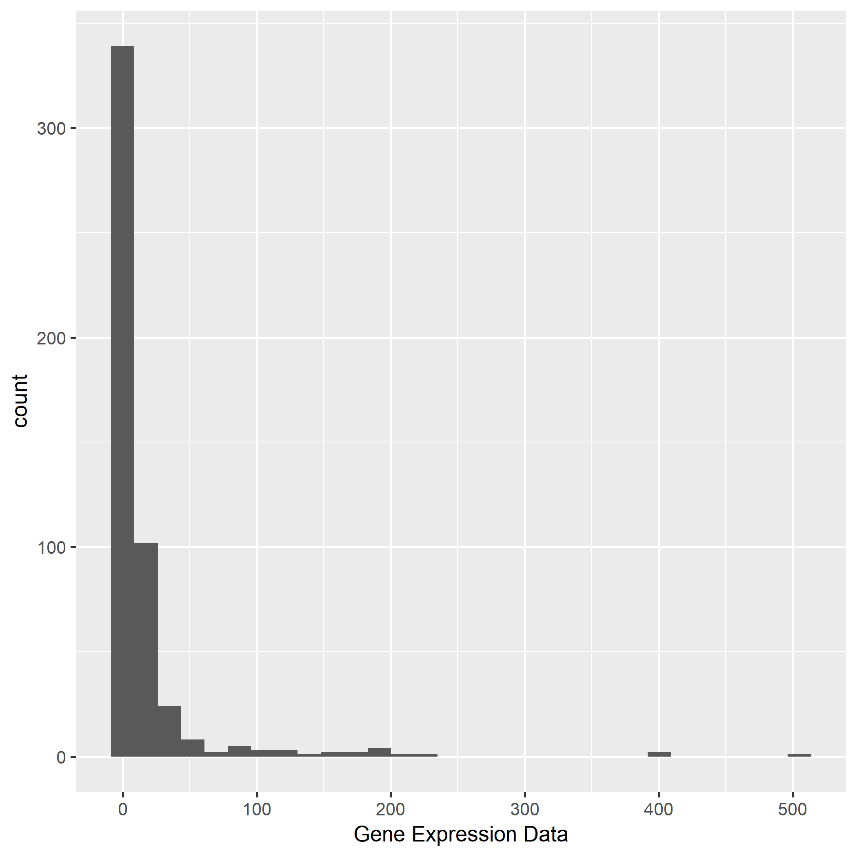


**Supplemental Table 1.** Simulation results are based on 100 replicates, including mean and standard deviation (SD) of the adjusted Rand index (ARI), false negative rate (FNR), false positive rate (FPR), and Matthews correlation coefficient (MCC). $\theta_{i}, \theta_{ni},\theta_{both}$ are the accuracy rates of informative feature weights, non-informative feature weights, and both informative and non-informative feature weights, respectively. Three simulation settings are considered. S1 spherical setting: $K=4, n=80, p_{1}=100, p_{2}=200$; S2 spherical setting: $K=4, n= 80, p_{1}=200, p_{2}=500$; S3 non-spherical setting with two half-moons: $K=2, n=80, p_{1}=100, p_{2}=200$. For S1 and S2, the cluster sizes are 10, 20, 40, 10, respectively, for four clusters. For S3, the cluster sizes are 20 and 60 for two clusters. The best ARI and MCC in each setting are in bold.

|  | Method | ARI | | FNR | | FPR | | MCC | |
| --- | --- | --- | --- | --- | --- | --- | --- | --- | --- |
|  |  | mean | SD | mean | SD | mean | SD | mean | SD |
| S1 spherical setting | iSCC ($\theta_{i}=1$) | 0.96 | 0.08 | 0.01 | 0.01 | <0.01 | <0.01 | **0.99** | 0.01 |
|  | iSCC ($\theta_{i}=0.7$) | 0.96 | 0.09 | 0.21 | 0.05 | 0.05 | 0.02 | 0.74 | 0.05 |
|  | iSCC ($\theta_{i}=0.5$) | 0.94 | 0.11 | 0.26 | 0.05 | 0.07 | 0.01 | 0.66 | 0.04 |
|  | iSCC ($\theta_{i}=0.3$) | 0.89 | 0.14 | 0.33 | 0.06 | 0.08 | 0.01 | 0.60 | 0.04 |
|  | iSCC ($\theta_{ni}=0.9$) | **0.98** | 0.05 | 0.11 | 0.09 | 0.02 | 0.02 | 0.88 | 0.05 |
|  | iSCC ($\theta_{ni}=0.8$) | 0.97 | 0.07 | 0.18 | 0.09 | 0.03 | 0.01 | 0.82 | 0.06 |
|  | iSCC ($\theta_{ni}=0.7$) | 0.97 | 0.08 | 0.23 | 0.09 | 0.03 | 0.01 | 0.77 | 0.06 |
|  | iSCC ($\theta_{both}=0.9$) | 0.96 | 0.09 | 0.12 | 0.04 | 0.04 | 0.02 | 0.82 | 0.05 |
|  | iSCC ($\theta_{both}=0.8$) | 0.97 | 0.08 | 0.24 | 0.07 | 0.07 | 0.04 | 0.68 | 0.07 |
|  | iSCC ($\theta_{both}=0.7$) | 0.90 | 0.14 | 0.35 | 0.06 | 0.09 | 0.05 | 0.57 | 0.09 |
|  | SCC | 0.61 | 0.09 | 0.28 | 0.03 | 0.09 | 0.01 | 0.61 | 0.02 |
|  | K-means | 0.61 | 0.14 | N.A. | N.A. | N.A. | N.A. | N.A. | N.A. |
|  | Sparse K-means | 0.55 | 0.04 | 0.34 | 0.02 | 0.09 | 0.02 | 0.57 | 0.05 |
|  | iCluster+ | 0.55 | 0.08 | 0.40 | 0.06 | 0.10 | 0.02 | 0.51 | 0.08 |
|  | BCC | 0.61 | 0.12 | N.A. | N.A. | N.A. | N.A. | N.A. | N.A. |
| S2 spherical setting | iSCC ($\theta_{i}=1$) | 0.98 | 0.04 | 0.03 | 0.04 | 0.01 | 0.01 | **0.95** | 0.02 |
|  | iSCC ($\theta_{i}=0.7$) | 0.98 | 0.05 | 0.21 | 0.04 | 0.02 | 0.01 | 0.76 | 0.04 |
|  | iSCC ($\theta_{i}=0.5$) | 0.96 | 0.08 | 0.28 | 0.05 | 0.03 | <0.01 | 0.69 | 0.04 |
|  | iSCC ($\theta_{i}=0.3$) | 0.90 | 0.12 | 0.31 | 0.04 | 0.03 | <0.01 | 0.66 | 0.03 |
|  | iSCC ($\theta_{ni}=0.9$) | **0.99** | 0.02 | 0.18 | 0.10 | 0.01 | <0.01 | 0.86 | 0.05 |
|  | iSCC ($\theta_{ni}=0.8$) | 0.97 | 0.08 | 0.22 | 0.09 | 0.01 | <0.01 | 0.83 | 0.04 |
|  | iSCC ($\theta_{ni}=0.7$) | 0.94 | 0.10 | 0.23 | 0.06 | 0.01 | <0.01 | 0.80 | 0.03 |
|  | iSCC ($\theta_{both}=0.9$) | 0.98 | 0.03 | 0.20 | 0.10 | 0.01 | 0.01 | 0.82 | 0.05 |
|  | iSCC ($\theta_{both}=0.8$) | 0.94 | 0.10 | 0.28 | 0.09 | 0.02 | 0.01 | 0.73 | 0.05 |
|  | iSCC ($\theta_{both}=0.7$) | 0.84 | 0.14 | 0.36 | 0.08 | 0.02 | 0.01 | 0.67 | 0.05 |
|  | SCC | 0.64 | 0.07 | 0.26 | 0.04 | 0.03 | <0.01 | 0.69 | 0.03 |
|  | K-means | 0.60 | 0.13 | N.A. | N.A. | N.A. | N.A. | N.A. | N.A. |
|  | Sparse K-means | 0.56 | 0.07 | 0.34 | 0.02 | 0.03 | 0.01 | 0.62 | 0.03 |
|  | iCluster+ | 0.56 | 0.11 | 0.64 | 0.05 | 0.02 | <0.01 | 0.43 | 0.07 |
|  | BCC | 0.56 | 0.11 | N.A. | N.A. | N.A. | N.A. | N.A. | N.A. |
| S3 non-spherical setting with two half-moons | iSCC ($\theta_{i}=1$) | **0.69** | 0.46 | 0.00 | 0.00 | 0.00 | 0.00 | **1.00** | 0.00 |
|  | iSCC ($\theta_{i}=0.7$) | 0.45 | 0.47 | 0.16 | 0.08 | 0.01 | 0.01 | 0.85 | 0.09 |
|  | iSCC ($\theta_{i}=0.5$) | 0.28 | 0.44 | 0.26 | 0.08 | 0.01 | 0.01 | 0.76 | 0.09 |
|  | iSCC ($\theta_{i}=0.3$) | 0.26 | 0.41 | 0.36 | 0.08 | 0.02 | 0.01 | 0.67 | 0.08 |
|  | iSCC ($\theta_{ni}=0.9$) | 0.63 | 0.39 | 0.02 | 0.06 | 0.35 | 0.43 | 0.53 | 0.29 |
|  | iSCC ($\theta_{ni}=0.8$) | 0.49 | 0.38 | 0.01 | 0.06 | 0.48 | 0.39 | 0.40 | 0.16 |
|  | iSCC ($\theta_{ni}=0.7$) | 0.51 | 0.34 | 0.01 | 0.03 | 0.71 | 0.36 | 0.25 | 0.19 |
|  | iSCC ($\theta_{both}=0.9$) | 0.61 | 0.39 | 0.05 | 0.07 | 0.58 | 0.45 | 0.32 | 0.28 |
|  | iSCC ($\theta_{both}=0.8$) | 0.63 | 0.32 | 0.04 | 0.07 | 0.76 | 0.37 | 0.24 | 0.18 |
|  | iSCC ($\theta_{both}=0.7$) | 0.36 | 0.34 | 0.05 | 0.08 | 0.75 | 0.34 | 0.22 | 0.13 |
|  | SCC | 0.10 | 0.26 | 0.04 | 0.13 | 0.10 | 0.08 | 0.64 | 0.15 |
|  | K-means | 0.28 | 0.06 | N.A. | N.A. | N.A. | N.A. | N.A. | N.A. |
|  | Sparse K-means | 0.61 | 0.08 | 0.02 | 0.10 | 0.06 | 0.06 | 0.73 | 0.15 |
|  | iCluster+ | 0.34 | 0.20 | 0.00 | 0.00 | 0.09 | 0.00 | 0.64 | 0.00 |
|  | BCC | 0.41 | 0.05 | N.A. | N.A. | N.A. | N.A. | N.A. | N.A. |

**Supplemental Table 2.** Selected features by iSCC from the TCGA breast cancer multi-omics dataset and the human lung cancer mRNA dataset

| 31 selected features by iSCC from the TCGA breast cancer multi-omics dataset | | | 18 selected features by iSCC from the human lung cancer mRNA dataset |
| --- | --- | --- | --- |
| mRNA | miRNA | Protein | mRNA |
| PGR | hsa-mir-1269 | PR | CHGA |
| MMP13 | hsa-mir-142 |  | SCG2 |
| NAT1 | hsa-mir-146a |  | TTR |
| S100P | hsa-mir-155 |  | IGHG4 |
| FOXA1 | hsa-mir-17 |  | SFTPC |
| AREG | hsa-mir-184 |  | PCK1 |
| S100A7 | hsa-mir-18a |  | ASCL1 |
| DKK1 | hsa-mir-203 |  | GRP |
| CP | hsa-mir-205 |  | SPP1 |
| S100A8 | hsa-mir-20a |  | CAV1 |
| ELF5 | hsa-mir-210 |  | IL6 |
| SFRP1 | hsa-mir-30a |  | MUC1 |
| ESR1 | hsa-mir-31 |  | ALDH1A1 |
| MIA | hsa-mir-335 |  | FN1 |
| AGR2 | hsa-mir-375 |  | PAM |
|  |  |  | CGA |
|  |  |  | NKX2-1 |
|  |  |  | CD74 |

**Supplemental Table 3.** Means of mRNA expression and p-values of t-test between iSCC identified clusters 1 and 2 for 18 selected genes for the human lung cancer mRNA dataset

| Gene id | mean of mRNA in cluster 1 | mean of mRNA in cluster 2 | p-value of t-test |
| --- | --- | --- | --- |
| CHGA | 3.25 | 4.26 | 3.33E-02 |
| SCG2 | 3.81 | 3.91 | 7.88E-01 |
| TTR | -0.72 | 5.76 | 3.29E-08 |
| IGHG4 | -4.35 | -2.96 | 4.35E-02 |
| SFTPC | -2.70 | -2.90 | 1.07E-01 |
| PCK1 | 0.79 | 4.13 | 4.50E-03 |
| ASCL1 | 2.89 | -0.92 | 1.64E-03 |
| GRP | 2.39 | 0.09 | 1.07E-01 |
| SPP1 | -0.19 | -1.59 | 7.99E-03 |
| CAV1 | -1.71 | -1.38 | 2.39E-01 |
| IL6 | -1.30 | -1.37 | 8.29E-01 |
| MUC1 | -1.85 | -1.96 | 4.54E-01 |
| ALDH1A1 | -1.73 | 2.47 | 6.00E-08 |
| FN1 | -1.79 | -2.00 | 6.40E-01 |
| PAM | 1.45 | 1.91 | 4.47E-01 |
| CGA | 2.11 | 1.18 | 3.93E-01 |
| NKX2-1 | 0.78 | -1.27 | 2.39E-04 |
| CD74 | -1.82 | -1.51 | 1.44E-01 |
